# The Effects of Postoperative Physician Phone Calls for Hand and Wrist Fractures: A Prospective, Randomized Controlled Trial

**DOI:** 10.7759/cureus.22202

**Published:** 2022-02-14

**Authors:** Scott N Loewenstein, Eric Pittelkow, Vasil V Kukushliev, Ivan Hadad, Joshua Adkinson

**Affiliations:** 1 Plastic and Reconstructive Surgery, Medical College of Wisconsin, Milwaukee, USA; 2 Plastic and Reconstructive Surgery, Indiana University School of Medicine, Indianapolis, USA

**Keywords:** postoperative care, wrist injuries, hand injuries, quality of care, patient satisfaction, patient compliance

## Abstract

Background

In this study, we sought to determine if postoperative physician phone calls following hand and wrist fracture surgery improve patient outcomes, satisfaction, and treatment adherence.

Methodology

We prospectively enrolled 24 consecutive adult patients who underwent outpatient surgery for isolated hand and wrist fractures at a single, metropolitan, safety-net hospital over one year to receive an additional physician phone call starting on postoperative day one. We measured preoperative and postoperative Brief Michigan Hand Questionnaire (bMHQ) composite score, overall satisfaction on a five-point Likert scale, compliance with treatment recommendations, presence of complications, discharge instructions reading level, and clarity of discharge and follow-up instructions. The surgical team was blinded to the treatment arm.

Results

The bMHQ score improved 26% after surgery; however, there was no difference in absolute score change between groups (12.2 vs. 6.5, p = 0.69). Most patients were satisfied throughout all stages of care, but postoperative satisfaction did not differ between groups (1.4 vs. 2.5, p = 0.21). There was a stronger correlation between patient hand function and satisfaction starting one month after surgery (R^2^ = 0.502, p = 0.002) than preoperatively (R^2 ^= 0.252, p = 0.029). Immediately following surgery, most patients stated that discharge instructions were clear, and the average readability was below the average patient education level. Despite this, 13% removed their splint or Kirschner wires, 67% did not follow up within a week of recommendation, 62% did not complete postoperative treatment, and 33% had complications.

Conclusions

Postoperative phone calls by physicians did not improve compliance with recommendations, patient-rated outcome measures, or clinical outcomes among our hand and wrist fracture patient population.

## Introduction

For patients with hand and wrist fractures, the effectiveness of operative treatment is closely related to adherence to postoperative instructions. Patients who are non-compliant with splinting recommendations are more likely to suffer from a non-union, decreased range of motion, increased pain, and overall decreased hand function [1-3]. Postoperative non-compliance rates among hand trauma patients can be high, ranging from 40% to 66% in some studies [4,5]. Patient adherence to a treatment plan is multifactorial and is associated with patient, provider, and system-related variables [6,7]. Across various clinical settings, strong physician-patient communication has been linked to patient adherence, treatment, and overall satisfaction [8-11].

Postoperative phone calls are a brief, low-resource intervention that can potentially improve outcomes for patients undergoing surgery for hand and wrist fractures. Furthermore, this simple intervention provides the opportunity to assess postoperative pain, review wound care instructions, and reiterate the details of the overall care plan. Previous research has shown that brief post-encounter phone calls improve patient satisfaction, symptoms, and adherence to treatment recommendations after discharge from hospitals and emergency departments [12,13]. Findings extend to surgical patients treated by urogynecologists, pediatric surgeons, trauma surgeons, and orthopedic surgeons [14-17]. Furthermore, trauma patients, when compared to non-trauma patients, view patient-physician communication as an area in need of improvement [18]. Thus, in this study, we sought to determine if postoperative physician phone calls reviewing discharge instructions improve outcomes among hand and wrist fracture patients. We hypothesized that this intervention would improve patient-reported hand function, overall patient satisfaction, adherence to treatment recommendations, and overall complication rates.

## Materials and methods

Study design

We performed a prospective, blinded, randomized controlled trial at a single urban midwestern level 1 trauma center. Consecutive patients with isolated fractures of the hand or wrist scheduled for outpatient operative treatment from January 2018 to December 2018 were included. We excluded patients who did not speak English, were less than 18 years of age, had trauma to other organ systems, or had open fractures. The study was approved by the Indiana University Institutional Review Board (IRB approval number: 1712309601) and registered on ClinicalTrials.gov. The study was conducted in accordance with the Helsinki Declaration of 1975.

Patients who provided informed consent completed a preoperative survey consisting of the brief Michigan Hand Questionnaire (bMHQ) [19], along with additional demographic questions and a question regarding overall satisfaction with care on a five-point Likert scale. Subsequently, patients underwent operative treatment and postoperative care based on the treating surgeon’s clinical judgment. All patients received personalized discharge instructions composed by the treating surgeon and read by their nurse to the patient before discharge. Upon discharge, patients were randomized to receive a phone call or not. To minimize bias, phone calls were performed by surgeons with the knowledge of hand surgery, but not directly involved in providing care to the patient.

Patients randomized to the treatment arm were called on postoperative day one. Patients were first asked to rate discharge instruction clarity, follow-up appointment time clarity, immobilization instruction clarity, and overall satisfaction of care on a five-point Likert scale. We then reviewed their discharge instructions and answered any related questions. Patients in all treatment arms were called no earlier than one month after surgery to repeat the bMHQ and answer additional questions about overall satisfaction with care.

Readability

Each surgeon composed his or her patient-specific postoperative instructions, which were reviewed with the patient by a nurse before discharge from the hospital. We calculated the Flesch-Kincaid reading score and grade level for each set of discharge instructions according to previously reported methods [20]. Scores for each group were averaged and compared for differences.

Statistical analysis

An a priori power analysis for the bMHQ composite score indicated that the study required the recruitment of 24 patients to detect a difference between groups (α = 0.05, power = 0.80). After completion of the study, results were grouped based on the treatment arm, and analysis was performed blinded to the identity of each treatment group. We first performed univariate analysis to obtain descriptive statistics. Subsequently, we performed bivariate analysis using chi-square and Fisher’s exact test for categorical variables and Student’s t-test for continuous variables. We performed a linear regression analysis to determine the correlation between variables and calculated the R^2^ statistic. An α of 0.05 was used to indicate statistical significance.

## Results

A total of 24 patients were included in the study. Patient characteristics are presented in Table 1. There were no differences between the two treatment arms. The majority of the patients were right-handed (70.8%), Black (58.3%), male (70.8%), and had an annual income of less than $30,000 (58.3%).

**Table 1:** Patient characteristics. SD: standard deviation

	No phone call	Phone call	All patients	
	(n = 14)	(n = 10)	(n = 24)	
	n (%)	n (%)	n (%)	P-value
Age (±SD) in years	39.3 (16.2)	34.9 (9.6)	37.5 (13.7)	0.452
Sex				1.000
Male	10 (71.4)	7 (70.0)	17 (70.8)	
Female	4 (28.6)	3 (30.0)	7 (29.2)	
Race				0.612
White	4 (28.6)	3 (30.0)	7 (29.2)	
Black or African American	7 (50.0)	7 (70.0)	14 (58.3)	
Asian	0 (0.0)	0 (0.0)	0 (0.0)	
Native Hawaiian or Pacific Islander	0 (0.0)	0 (0.0)	0 (0.0)	
American Indian or Alaskan native	0 (0.0)	0 (0.0)	0 (0.0)	
Other	2 (14.3)	0 (0.0)	2 (8.3)	
Choose not to specify	1 (7.1)	0 (0.0)	1 (4.2)	
Highest degree				0.891
Less than high school graduate	3 (21.4)	1 (10.0)	4 (16.7)	
High school graduate	7 (50.0)	7 (70.0)	14 (58.3)	
Vocational/technical school	0 (0.0)	0 (0.0)	0 (0.0)	
Some college or associate degree	2 (14.3)	1 (10.0)	3 (12.5)	
College graduate	0 (0.0)	0 (0.0)	0 (0.0)	
Professional or graduate school	1 (7.1)	0 (0.0)	1 (4.2)	
Income				0.151
<10k	4 (28.6)	2 (20.0)	6 (25.0)	
<20k	2 (14.3)	3 (30.0)	5 (20.8)	
<30k	0 (0.0)	3 (30.0)	3 (12.5)	
<40k	2 (14.3)	0 (0.0)	2 (8.3)	
<50k	0 (0.0)	1 (10.0)	1 (4.2)	
<60k	1 (7.1)	0 (0.0)	1 (4.2)	
<70k	0 (0.0)	0 (0.0)	0 (0.0)	
≥70k	0 (0.0)	0 (0.0)	0 (0.0)	
Handedness				1.000
Right hand	9 (64.3)	8 (80.0)	17 (70.8)	
Left hand	2 (14.3)	1 (10.0)	3 (12.5)	
Ambidextrous	2 (14.3)	1 (10.0)	3 (12.5)	

Analysis of composite bMHQ scores and overall patient satisfaction is presented in Table 2. There were trends toward improved bMHQ scores in both groups after surgery and improved overall satisfaction in the postoperative phone call group; however, these were not statistically significant. Most patients were “satisfied” or “somewhat satisfied” with their care preoperatively (89.5%), immediately postoperatively (85.7%), and late postoperatively (73.3%). There was a stronger correlation between patient hand function, as measured by bMHQ scores, and satisfaction with care starting one month after surgery (R^2^ = 0.502, p = 0.002) compared to preoperatively (R^2^ = 0.252, p = 0.029).

**Table 2:** Patient-reported function and satisfaction before and after surgery. ­P-values for comparisons between treatment arms are ^a^0.617, ^b^0.826, ­^c^0.615, and ^d^0.208. bMHQ: Brief Michigan Hand Questionnaire; SD: standard deviation

	No phone call			Phone call		
	Preoperative	Postoperative	P-value	Preoperative	Postoperative	P-value
Mean bMHQ score (SD)	52.48 (13.95)	61.43 (17.81)	0.221	49.67 (12.47)^a^	58.75 (26.72)^b^	0.352
Mean satisfaction score (SD)	1.50 (0.85)	1.43 (1.13)	0.884	1.33 (0.50)^c^	2.50 (1.85)^d^	0.150

The average readability of all discharge instructions was grade 7.69 (±2.56), and it did not vary between patients who received phone calls (7.36 ± 2.60) and those who did not (8.04 ± 2.68, p = 0.47).

Of the patients randomized to the postoperative phone call group, six were successfully contacted starting on postoperative day one to query their understanding of discharge instructions and review them. All these patients felt that discharge instructions were somewhat or very clear, 83% felt that follow-up appointment time was clear, and 83% felt that immobilization instructions were somewhat or very clear. Despite this, 12.5% of patients removed their own splint or Kirschner wires, 66.6% did not follow up within one week of recommendation, and 62.5% did not complete the postoperative treatment recommendations to be satisfactorily discharged from care (Table 3). One-third of patients had complications, which included pin site infections, bleeding, delayed wound healing, and pain necessitating emergency room visit.

**Table 3:** Patient outcomes.

	No Phone Call	Phone Call		All patients
	n (%)	n (%)	P-value	n (%)
Pin site infection	0 (0.0)	2 (20.0)	0.163	2 (8.3)
Bleeding complication	1 (7.1)	0 (0.0)	1.000	1 (4.2)
Delayed wound healing	1 (7.1)	0 (0.0)	1.000	1 (4.2)
Emergency room visit	3 (21.4)	2 (20.0)	1.000	5 (20.8)
Self-removal of splint or hardware	2 (14.3)	1 (10.0)	1.000	3 (12.5)
Follow-up within seven days of recommendation	4 (28.6)	4 (40.0)	0.673	8 (33.3)
Completed treatment and discharged	4 (28.6)	5 (50.0)	0.403	9 (37.5)

## Discussion

In this study, we found no evidence that a postoperative physician phone call reviewing discharge instructions improves outcomes among hand trauma patients who undergo outpatient surgery at a large, urban, safety-net hospital. Specifically, postoperative physician phone calls did not increase patient satisfaction, decrease complications, or improve patient-reported hand function. We found a significant non-compliance rate among our hand fracture population, with many patients prematurely discontinuing their splints, removing their Kirschner wires, and not attending the recommended postoperative follow-up appointments. This is despite most patients expressing understanding treatment recommendations during their postoperative survey.

The causes of non-compliance are complex and multifactorial. When it leads to a poor outcome, it may be difficult for the provider to remain non-judgemental [21]. Nevertheless, patients may be in situations where they are forced to choose between non-adherence with care and a perceived or real negative consequence related to following through with physician recommendations. The majority of patients in this study were below the federal poverty line. A patient who cannot afford transportation or taking time off work may not be able to attend frequent therapy visits or physician follow-up appointments.

Attempts to improve adherence among hand trauma patients with home instructional videos and/or adjusting the type of postoperative immobilization have been met with varying success [4,22]. We found that postoperative physician phone calls, however, did not increase patient adherence in this patient population. To address the high non-compliance rates, treatment should be catered to the patients’ circumstances, whenever possible. For example, a non-removable cast can be used in place of a splint, hardware can be buried to decrease inadvertent removal and infection risk, and follow-up frequency can be adjusted to accommodate the patient’s schedule. By identifying patient-specific barriers and opportunities for improvement, we can target treatment specifically to improve outcomes [23]. This may have the beneficial side effect of decreasing provider burnout related to the feeling of helplessness regarding improving outcomes in patients with challenging socioeconomic circumstances.

With hospital and physician remuneration increasingly being tied to patient satisfaction, the patient experience has also become a frequent measure of the quality of care. Although Gotlib Conn et al. identified poor information exchange upon discharge among trauma patients and unavailability of surgeons as two detriments to quality, we found that postoperative physician phone calls do not increase patient satisfaction of care among hand fracture patients [24]. Many patients who knew that there was a significant chance that they would be called by a physician after surgery did not answer their phone, even after multiple attempts and perioperative confirmation with the patient of the correct contact information. This finding suggests physician-initiated contact is not as important to patients as physician availability during patient-initiated contact. Hence, promoting minimally impeded access to physicians to answer questions may improve patient satisfaction in this population; however, this must be balanced against other demands on surgeon time.

A potential source for non-adherence with treatment and the development of complications is the lack of understanding of postoperative instructions. We explored the readability of patient discharge instructions as a potential area to improve patient compliance. Although the readability of patient discharge instructions was below the average readability for other hand surgery resources, they were still above the sixth-grade reading level recommended by the National Institutes of Health, the American Medical Association, and the US National Library of Medicine [25-27]. In this study population, however, the majority of patients had at least completed high school and were expected to have the literacy skills to interpret the discharge instructions their surgeons provided them. Furthermore, when surveyed starting the day after surgery, most patients reported that discharge instructions were clear. These findings would suggest that a lack of understanding of postoperative instructions is not an important part of the mechanism for patient non-compliance.

Although postoperative phone calls did not result in a statistically significant improvement in overall patient satisfaction, we did find a direct correlation between postoperative patient satisfaction and patient-reported hand function. This finding suggests that among patients with hand and wrist fractures, postoperative satisfaction is strongly weighted by patients’ ability to use their hands and less by their experience. Therefore, future aims at improving quality should primarily target optimizing postoperative hand function.

This study has several limitations. First, the number of patients who did not answer their phone after surgery was higher than expected, which decreased statistical power. Second, this study did not measure physician empathy, which has been demonstrated to be a confounder for patient satisfaction among hand surgery patients. Among hand surgery patients in the outpatient setting, for example, physician empathy independently accounted for 34% to 65% of patient satisfaction [28,29]. A postoperative phone call that focused on empathetic listening, rather than reviewing discharge instructions, may have been more beneficial to increasing patient satisfaction. Additionally, this study focused on a specific population of patients, and generalizability to all patients undergoing hand surgery should be approached with caution. Finally, although the Flesch-Kincaid formula has been validated, any formula to objectively measure readability does not assess patients’ true understanding of the written text and therefore is only an estimate of comprehension [25,30]. With these caveats in mind, this study has successfully demonstrated that a postoperative physician phone call reviewing discharge instructions is not an effective intervention to improve quality in this patient population.

## Conclusions

Non-adherence to treatment recommendations is prevalent among certain populations treated for hand and wrist fractures. It affects quality and represents a clinically significant aspect of hand surgery that affects outcomes. Although patient-physician communication has been identified by patients as an area needing improvement, postoperative physician-initiated phone calls did not improve patient-defined aspects of quality after treatment for hand and wrist fractures. Improving postoperative physician access for patient-initiated phone calls and adapting treatment plans to address unique patient barriers may prove to have a greater impact on quality.
